# TRPS1 Confers Multidrug Resistance of Breast Cancer Cells by Regulating BCRP Expression

**DOI:** 10.3389/fonc.2020.00934

**Published:** 2020-06-30

**Authors:** Jing Hu, Hui Zhang, Long Liu, Bo Han, Gengyin Zhou, Peng Su

**Affiliations:** Department of Pathology, Qilu Hospital of Shandong University, Jinan, China

**Keywords:** TRPS1, BCRP, chemotherapy, multidrug resistance, breast cancer

## Abstract

Multidrug resistance (MDR) is the major obstruction in the successful treatment of breast cancer (BCa). The elucidation of molecular events that confer chemoresistance of BCa is of major therapeutic importance. Several studies have elucidated the correlation of TRPS1 and BCa. Here we focused on the role of TRPS1 in acquisition of chemoresistance, and reported a unique role of TRPS1 renders BCa cells resistant to chemotherapeutic drugs via the regulation of BCRP expression. Bioinformation analysis based on publicly available BCa data suggested that TRPS1 overexpression linked to chemoresistance. Mechanistically, TRPS1 regulated BCRP expression and efflux transportation. Overexpression of TRPS1 led to upregulation of BCRP while its inhibition resulted in repression of BCRP. The correlation of TRPS1 and BCRP was further confirmed by immunohistochemistry in 180 BCa samples. MTT assay demonstrated that manipulation of TRPS1 expression affects the chemosensitivity of BCa cells. In total, high expression of TRPS1 confers MDR of BCa which is mediated by BCRP. Our data demonstrated a new insight into mechanisms and strategies to overcome chemoresistance in BCa.

## Introduction

To date, breast cancer (BCa) ranks first among female malignant tumor, accounting for 30%, and the second leading cause of cancer mortality in women, accounting for 15% (1). Surgery is the predominant treatment of BCa. Chemotherapy may be used as adjuvant therapy to shrink the tumor before surgery or to prevent remission/relapse after surgery (2). Multidrug resistance (MDR) is a major limitation for chemotherapy leading to treatment failure and tumor relapse (3, 4). Hence, overcoming chemoresistance is of paramount scientific and therapeutic importance in medical oncology (5).

Mechanisms of drug resistance include mutations of drug targets, alterations in drug metabolism, increased efflux of anticancer drugs, and the activation of survival or inactivation of downstream death signaling pathways (6–8). The efflux transportation was carried out by ATP-binding cassette (ABC) transporters which transport substrate drugs out of the cell against a concentration gradient in energy dependent manner (7). Breast cancer resistance protein (BCRP) belongs to the subfamily of G of the ABC transporter superfamily and has been extensively studied. BCRP overexpresses in many cancer cell lines and can efflux a variety of chemotherapeutic agents including adriamycin, epirubicin, mitoxantrone, topotecan, methotrexate, doxorubicin, and flavopiridol out of the cell (9–11). Several studies have focused on potent inhibitor of BCRP to specifically block the drug efflux activity (12).

The TRPS1 transcription factor is encoded by the *TRPS1* gene, of which deletions and mutations cause the tricho-rhino-phalangeal syndromes. To date, increased evidence suggested TRPS1 involved in a wide variety of functions among human malignancies including BCa and prostate cancer (13, 14). We and others showed that TRPS1 suppresses epithelial-mesenchymal transition (EMT) as a tumor suppressor (15–17). High expression of TRPS1 correlated with decreased metastasis and better prognosis. However, the bioinformatics analysis of publically available BCa dataset showed that high TRPS1 expression is associated with increased metastasis and poor prognosis in patients received chemotherapeutics. Therefore, it is of interest to investigate whether TRPS1 plays a role in acquisition of chemoresistance.

## Materials and Methods

### Bioinformatics Analysis

Datasets of GSE45255, GSE25055, GSE12791, and GSE27830 were downloaded from the Gene Expression Omnibus (GEO) database (http://www.ncbi.nlm.nih.gov/geo). The online tool Kaplan-Meier Plotter was also used for survival analysis (http://kmplot.com/analysis/) (18). The online tool R2 was also used for correlation analysis (R2: Genomics Analysis and Visualization Platform, http://r2.amc.nl). GSE114213 was obtained from Cistrome (http://cistrome.org/db/#/) and visualized by UCSC (http://genome.ucsc.edu/index.html).

### Cell Lines and Cell Culture

The BCa cell lines MDA-MB-231 and MCF-7 were purchased from American Type Culture Collection (Manassas, VA, USA). MDA-MB-231 cells were cultured in Leibovitz's L-15 (Gibco, Grand Island, NY, USA) Medium containing 10% fetal bovine serum. MCF-7 cells were maintained in Dulbecco's modified Eagle's (Gibco) medium containing 0.01 mg/ml bovine insulin and 10% fetal bovine serum. Both were at 37°C in a humidified incubator supplied with 5% CO_2_.

### Plasmids and Transfection

To create TRPS1 plasmids, sequences for TRPS1 (MHS6278-211690440, Thermo Fisher Scientific, Lafayette, USA) were cloned into a mammalian expression vector, pcDNA3.1 (+). TRPS1-specific shRNA was purchased from Shanghai Gene Pharma Co., Ltd. shRNA oligonucleotide sequences targeted at TRPS1 were as follows: forward,5′-CACCGCTGCAGAACTAAATCATAAGTTCAAGAGACTTATGATTTAGTTCTGCAGCTTTTTTG-3′; reverse,5′-GATCCAAAAAAGCTGCAGAACTAAATCATAAGTCTCTTGAACTTATGATTTAGTTCTGCAGC-3′. MDA-MB-231 cells transfected with vectors are referred as 231-Vec and 231-TRPS1, and MCF-7 cells transfected with shRNA are referred as MCF7-nc and MCF7-TS.

TurboFectTM (Fermentas, Burlington, Canada) was used to stably transfect BCa cells.

### RNA Isolation and Real-Time PCR Analysis

Total RNA was isolated from cultured cells using RNAiso Plus (Takara, Dalian, China), and the complementary DNA was synthesized using ReverTra Ace qPCR RT Master Mix (TOYOBO, Osaka, Japan) both according to the manufacturers' instructions. mRNA was quantified by real-time PCR analysis using UltraSYBR Mixture with ROX (Beijing CoWin Biotech Co., Ltd., China) and ΔΔCt method. β-actin was used as reference gene. The following primer pairs were used for real-time PCR analysis:

TRPS1 forward primer, 5′-CAAATCTCAGGCCTGAGTGA-3′, reverse primer, 5′-GTGAAGAGCTGATATCCTGCAG-3′;

BCRP forward primer, 5′-TGGCTGTCATGGCTTCAGTA-3′, reverse primer, 5′-GCCACGTGATTCTTCCACAA-3′,

β-actin forward primer 5′-CTCCATCCTGGCCTCGCTGT-3′, reverse primer 5′-GCTGTCACCTTCACCGTTCC-3′.

### Western Blot Analysis

Cellular protein extracts were prepared as described previously (19). The membranes were incubated with antibodies overnight at 4°C. Immunoreactivity was detected using an enhanced chemiluminescence kit (Millipore, Darmstadt, Germany).

The primary antibodies were as follows: goat polyclonal antibody TRPS1 (diluted 1:500, Santa Cruz), mouse anti-BCRP (BXP21) (diluted 1:200, Abcam) and mouse mAb beta-actin (diluted 1:2,000, TA-09; Zhongshan Golden bridge Biotechnology, Beijing, China) as a total protein internal control.

### Cytotoxicity Assay

The 3-(4.5-dimethylthiazed-2-yl)-2,5-diphenylterazolium bromide (MTT, Sigma, San Francisco, CA, USA) assay was used to assess the chemosensitivity of selected transfection cells to anticancer drugs. The cells were plated in a 96-well plate at a density of 8 × 10^3^ cells per well for 24 h and then incubated with different concentrations of doxorubicin, cisplatin, paclitaxel, 5-Fu (Dalian Meilunbio Co., Ltd., Dalian, China), and mitoxantrone (Selleckchem, Houston, TX, USA) for 48 h. The MTT assay was performed following the manufacturer's instructions. Absorbance was measured using a spectrometric absorbance of 570 nm against a background of 630 nm on a Bio-Rad microplate reader (Hercules, CA, USA). The dose-dependent curve and 50% inhibitory concentration (IC50) were calculated to determine the chemosensitivity of the cells.

### Intracellular Mitoxantrone Efflux Assay

Mitoxantrone, a known high-affinity substrate of BCRP, was used to detect BCRP transport activity. Approximately 1 × 10^6^ cells were incubated with 25 μM mitoxantrone for 30 min and then allowed to efflux for 2 h in complete medium alone. The cells were washed twice with ice-cold PBS and then resuspended in 500 μl of serum-free medium. All cells were analyzed by flow cytometry (BD AccuriC6, San Jose, CA, USA) with a 635-nm red diode laser and 670-nm band-pass filter. The fluorescence intensity of control group was set as background to exclude non-specific fluorescence. Data were analyzed with FlowJo 7.6.5 (Stanford University, California, USA).

### Patients and Tissue Samples

Paraffin-embedded samples from 180 patients with invasive ductal BCa were analyzed at the Department of Pathology, Qilu Hospital of Shandong University from 2007 to 2009. Patient consent and approval from the Research Ethics Committee of Shandong University School of Medicine were obtained.

### Immunohistochemistry

Immunohistochemistry was performed as described in previously (20). The slides were incubated in primary antibodies to TRPS1 (sc-26974, diluted 1:200; Santa Cruz Biotechnology, CA, USA) and BCRP (clone BXP21, diluted 1:40, Abcam, Cambridge, MA, USA), overnight at 4°C. Sections were stained with diaminobenzidine (DAB) and counterstained with hematoxylin. For negative controls, the antibodies were replaced with PBS.

### Evaluation of Immunohistochemical Staining

The stained slides were evaluated independently by two independent pathologists (S.P and H.J.) based on previously described semi-quantitatively scoring system. The TRPS1 expression was scored by the product of intensity (0 = negative; 1 = weak; 2 = moderate; and 3 = strong) and percentage of extent of reactivity (0 = 0–10%; 1 = 20–30%; 2 = 30–50%; 3 = 50–80%; and 4 = 80–100%). The following cut-off levels were applied: 0 for negative, 1–4 for weak positive, and 6–12 for strong positive (21). BCRP expression was scored for intensity (0 = negative; 1 = weak; 2 = moderate; 3 = strong) and the percentage of positive cells (0 = 0–10%; 1 = 10–40%; 2 = 40–70%; 3 = 70–100%). The optimal cut-off value for high and low expression level was identified: 0 for negative, 1–3 for low BCRP expression, and 4–9 for high expression (22, 23).

### Statistical Analysis

Statistical analysis was performed using SPSS 22.0 (SPSS, Chicago, IL, USA) and Graph Pad Prism 7 (Graph Pad Software, Inc., San Diego, CA, USA). All data were presented as mean ± SEM, with *p* < 0.05 considered as statistically significant. Correlation significance was assessed with Pearson Chi-square test or Fisher's exact test if appropriate.

## Result

### TRPS1 Expression Is Related to Chemoresistance in BCa

**Figure 1:**
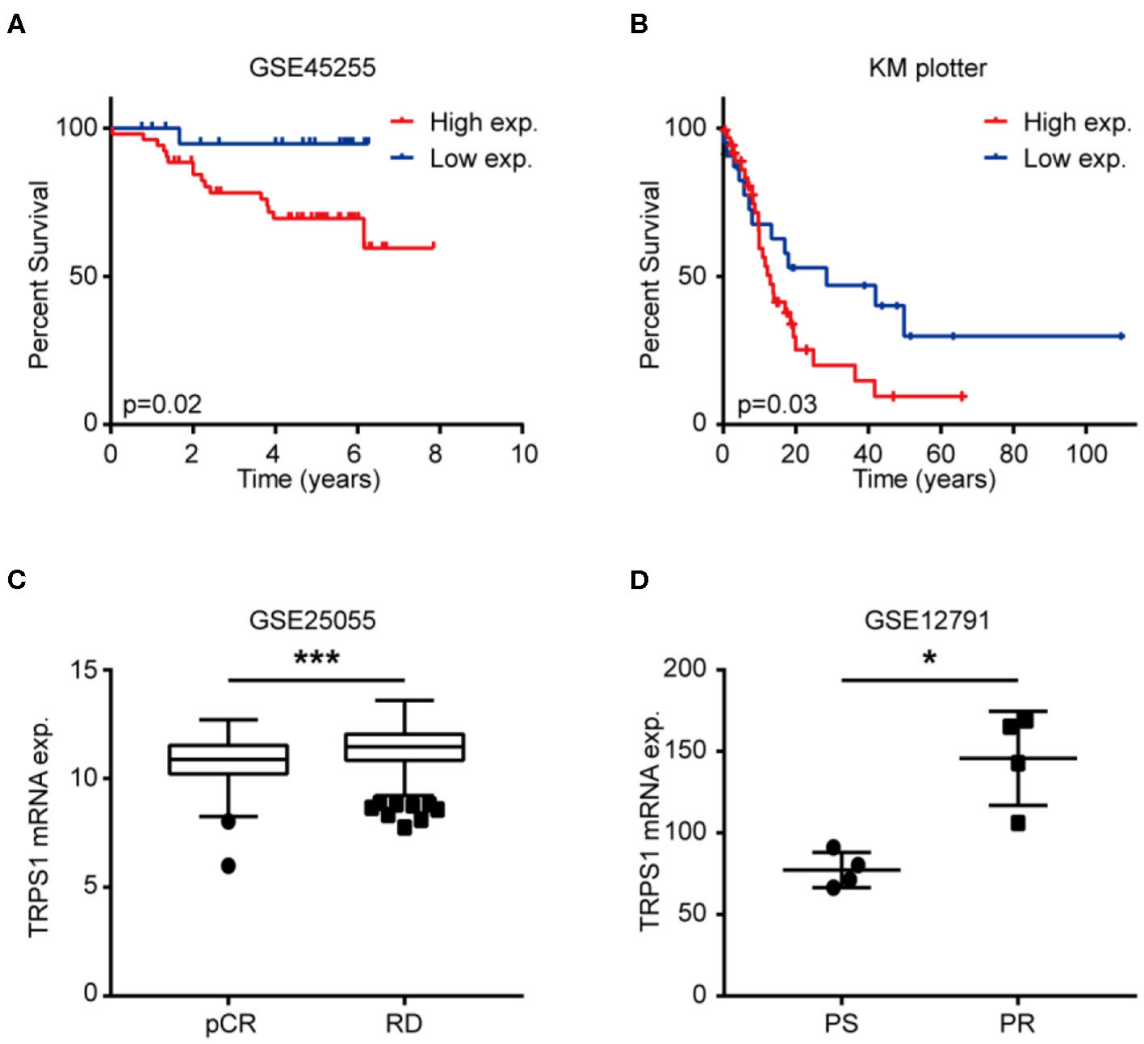
TRPS1 expression was related with poor prognosis after chemotherapy and chemoresistance of BCa. **(A,B)** Kaplan–Meier survival analysis of BCa cases with chemotherapy from GSE45255 (**A**, *p* = 0.02, Log-rank test) and KM plotter cohort (**B**, *p* = 0.03, Log-rank test) according to high and low TRPS1 expression. **(C)** Expression of TRPS1 in BCa tissues with pCR compared with BCa tissues with RD in GSE25055. pCR, pathological complete response; RD, residual disease. **(D)** Expression of TRPS1 in paclitaxel sensitive (PS) and paclitaxel resistant (PR) BCa cell driven from MDA-MB-231 in GSE12791. *P < 0.05, ***P < 0.001.

Several studies have demonstrated patients with high expression of TRPS1 displayed a lower rate of metastasis. To explore the relationship of TRPS1 expression and prognosis in BCa patients after chemotherapy, Kaplan–Meier plotting and log-rank test were carried out with publicly accessible datasets. Of note, patients after chemotherapy with higher TRPS1 expression exhibited worse distant metastatic overall survival (Figure 1A, GSE45255, *p* = 0.02) and overall survival (Figure 1B, KM plotter, *p* = 0.03), respectively. To further investigate the association of TRPS1 expression and chemoresistance, TRPS1 mRNA expression was analyzed using publicly accessible gene expression data containing chemosensitive and chemoresistant samples. As shown in Figure 1, we found that TRPS1 expression was significantly increased in chemoresistant patients (Figure 1C, GSE25055, *p* < 0.0001), and chemoresistant BCa cells (Figure 1D, GSE12791, *p* = 0.015).

### TRPS1 Induced BCRP Expression of BCa

**Figure 2:**
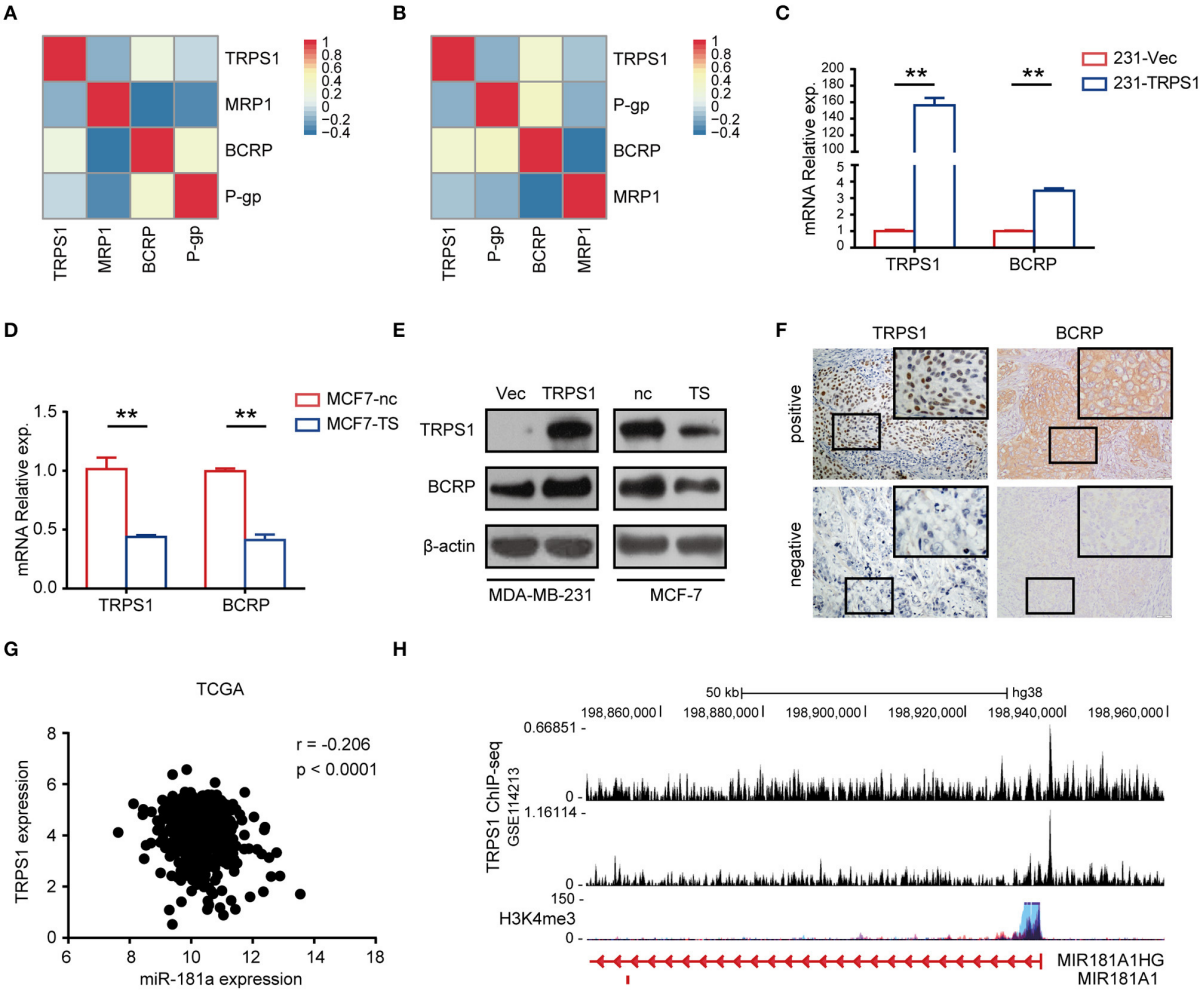
TRPS1 regulated BCRP expression. **(A,B)** Correlation of TRPS1 and ABC transporters in R2 database **(A)** and GSE27830 **(B)**. **(C,D)** qRT-PCR analyses of TRPS1 and BCRP mRNA expression of indicated cells (**C**, 231-Vec/231-TRPS1; **D**, MCF7-nc/MCF7-TS). **(E)** Western blots analyses of TRPS1 and BCRP protein expression of indicated BCa cell lines transfected with nc/TS or Vec/TRPS1. **(F)** Representative images of IHC staining of TRPS1 and BCRP in BCa patients. **(G)** Correlation of TRPS1 and miR-181a in TCGA data. **(H)** Enrichment of TRPS1 at miR-181a promotor region determined by ChIP-seq experiment (GSE114213). **P < 0.01.

To explore the mechanisms of TRPS1 in chemoresistance, we analyzed the expression of TRPS1 and several ABC transporters, including BCRP, P-glycoprotein (P-gp), and multidrug resistance associated protein (MRP1). Although TRPS1 has been associated with P-gp expression in osteosarcoma (24), the expression of TRPS1 did not show positive correlation with P-gp in pancancer dataset (Figure 2A, R2, *n* = 40103) and BCa dataset (Figure 2B, GSE27830, *n* = 155). TRPS1 expression showed positive correlation to BCRP expression (Figure 2A, R2, pancancer, *p* < 0.0001, *r* = 0.018; Figure 2B, GSE27830, BCa dataset, p = 0.003, r = 0.234). To validate the relationship between TRPS1 and BCRP, we examined BCRP expression in BCa cells after alteration of TRPS1 expression. Compared to control cells (231-Vec), an average of 3.5-fold higher BCRP mRNA expression was observed by qRT-PCR in 231-TRPS1 cells (Figure 2C, *p* < 0.01). In the meantime, the relative expressions of BCRP mRNA in MCF7-TS cells were 4.1-fold lower than that of the control cells (Figure 2D, *p* < 0.01). Results of western blot analysis for BCRP proteins showed the same trend as that of qRT-PCR analysis (Figure 2E). To further confirm whether TRPS1 and BCRP expression is physiologically relevant in BCa patients, 180 samples were included for IHC analysis. As shown in Figure 2F, the expression of TRPS1 and BCRP was heterogeneous within the tumors. Ninety-three of 180 patients were TRPS1 positive (51.67%), and 110 of 180 were BCRP positive (61.11%). Statistical analyses indicated that the immunohistochemical expression of TRPS1 directly correlated with BCRP (*p* = 0.012). These results were further confirmed by Spearman correlation analysis (Table 1, *r* = 0.186, *p* = 0.012). It has been documented that miR-181a regulated BCRP expression via binding to the 3′-UTR of BCRP mRNA (25). Bioinformatics analysis suggested that TRPS1 was negative related with miR-181a (Figure 2G) and enriched in the promotor region of miR-181a (Figure 2H).

**Table 1:** Correlation between TRPS1 expression and BCRP expression in breast cancer.

**Characteristics**	***N***	**TRPS1 expression**		***P-*value**	**Spearman**	**Value (r)**	***P-*value**
		**Negative**	**Positive**		**Correlation**		
BCRP							
≤3	118	64	54	0.029		0.163	0.029
>3	62	23	39				

*The Spearman correlation was used to compare the degrees of correlation. Positive numbers reflected direct correlation, and negative numbers reflected inverse correlation*.

### Effects of TRPS1 on BCRP-Mediated Mitoxantrone Efflux Activity

**Figure 3:**
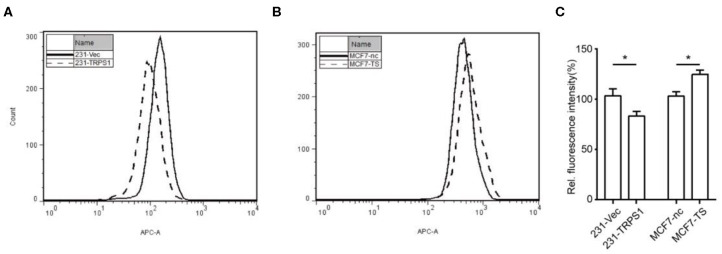
Mitoxantrone efflux assay for determining the effects of TRPS1 on the BCRP function. The BCRP efflux function of breast cancer cells was measured by flow cytometry. **(A)** BCRP function as an efflux pump was activated remarkably in TRPS1-transfected breast cancer cells compared to controls. **(B)** BCRP function as an efflux pump decreased remarkably in TRPS1-silenced breast cancer cells compared to controls. **(C)** Intracellular fluorescence intensity analysis was performed, and representative data from three independent experiments are shown. *P < 0.05.

The functional efflux assay was based on the extrusion of mitoxantrone, high-affinity substrate of BCRP. Compared to 231-Vec cell, 231-TRPS1 cells exhibited rapid reduction in intracellular mitoxantrone (Figure 3A). We also noticed a rightward shift in the fluorescence peak in MCF7-TS cells, which means deceased efflux of mitoxantrone according to TRPS1 inhibition (Figure 3B). Similar results were found in repeated experiments. Intracellular fluorescence intensity was shown in Figure 3C. These results demonstrated that TRPS1 in fact influences BCRP efflux function.

### Overexpression of TRPS1 Renders Cells Resistant to Chemotherapeutic Drugs

**Figure 4:**
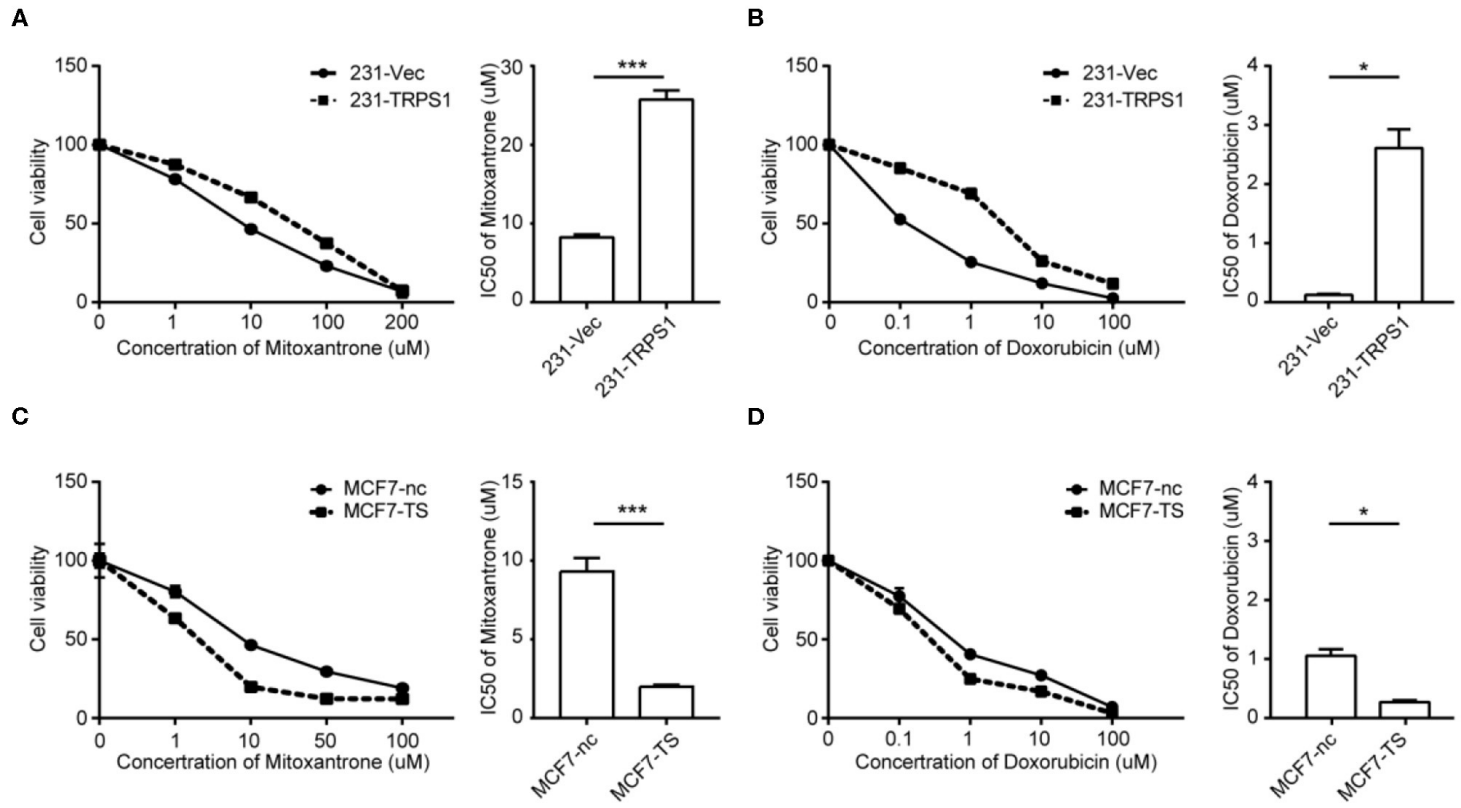
TRPS1 correlated with drug sensitivity of BCa cells. **(A,B)** MDA-MB-231 cells were transfected with plasmids expressing TRPS1 (231-TRPS1) or empty plasmid (231-Vec). **(C,D)** MCF-7 cells were transfected with siRNA-targeting TRPS1 (MCF7-TS) or negative control (MCF7-nc). Cell viability and 50% inhibitory concentration (IC50) were assessed using the MTT assay after treatment of chemotherapeutic drugs. Cell viability was calculated relative to untreated controls. **(A,C)** mitoxantrone; **(B,D)** doxorubicin. Left panel: Cell viability. Right panel: 50% inhibitory concentration (IC50). Data represent averages from triplicates in a representative experiment, and their standard errors are depicted. *P < 0.05, ***P < 0.001.

We overexpressed TRPS1 in MDA-MB-231 cells to determine whether TRPS1 affects chemoresistance in BCa cells. Overexpression of TRPS1 in MDA-MB-231 cells (231-Vec/231-TRPS1) was verified by western blot (Figure S1A). The selected cells were treated with mitoxantrone, doxorubicin, cisplatin, 5-Fu, and paclitaxel at different concentrations for 48 h, and cytotoxicity was examined by MTT assay. As shown in Figures 4A,B, TRPS1 overexpression dramatically increased the IC50 values of mitoxantrone from 8.21 ± 0.808 μM to 25.66 ± 3.974 μM and doxorubicin from 0.13 ± 0.02 μM to 2.61 ± 0.32 μM, respectively (Table 2). We also found increased IC50 values of paclitaxel, cisplatin, and 5-Fu after TRPS1 overexpression (Figures S2A,C, Table 2).

**Table 2:** The IC50 values of the four chemotherapeutic drugs in different groups of cells.

**Cells**	**Drugs**				
	**Mitoxantrone (μM)**	**Doxorubicin (μM)**	**Cisplatin (μg/mL)**	**Paclitaxel (ng/mL)**	**5-Fu (mM)**
231-Vec	8.21 ± 0.808	0.125 ± 0.0167	1.22 ± 0.0612	1.31 ± 0.153	0.134 ± 0.00297
231-TRPS1	25.66 ± 3.974	2.61 ± 0.317	2.73 ± 0.268	3.18 ± 0.293	0.396 ± 0.0382
MCF7-nc	9.326 ± 0.619	1.05 ± 0.113	4.54 ± 0.231	7.35 ± 0.188	0.204 ± 0.00983
MCF7-TS	1.992 ± 0.288	0.272 ± 0.0289	1.62 ± 0.0457	2.95 ± 0.4	0.099 ± 0.0091

*The IC50 values of mitoxantrone doxorubicin, cisplatin, paclitaxel, and 5-Fu were averaged from triplicates of MTT assays with GraphPad Prism 5, presented as mean ± SEM. IC50 values of the four anticancer drugs were all increased after overexpression of TRPS1 in MDA-MB-231 cells. Furthermore, the decrease could be observed by TRPS1 silence in MCF7 cells*.

### Silencing TRPS1 Inhibits Chemoresistance of BCa Cells

Inhibition of TRPS1 in MCF-7 cells (MCF7-ns/MCF7-TS) was verified by western blot (Figure S1B). The selected cells were subjected to chemotherapeutic treatment at different concentrations for 48 h, and cytotoxicity was examined by MTT assay. As shown in Figures 4C,D, TRPS1 inhibition dramatically decreased the IC50 values of mitoxantrone from 9.326 ± 0.619 μM to 1.992 ± 0.288 μM and doxorubicin from 1.05 ± 0.11 μM to 0.27 ± 0.03 μM, respectively (Table 2). The IC50 values of paclitaxel, cisplatin, and 5-Fu also decreased after TRPS1 inhibition (Figures S2D,F, Table 2).

## Discussion

The rapid development of chemoresistance is the major obstacle in the effective treatment of BCa. One of the mechanisms of MDR is the elevated expression of ABC transporters. The ABC transporters increase transmembrane efflux of drugs from cells that causes the decrease of their concentration in cytoplasm and, in consequence of that, insensitivity of cell to damaging impact of cytostatics (26). BCRP is one of three primary types of ABC transporters in human relating to the MDR phenomenon, and the other two are P-gp and MRP-1.

BCRP, also known as the ATP-binding cassette (ABC) transporter G2 (ABCG2) (10, 27), is designated as a half transporter and has the function of efflux various structurally and functionally unrelated anticancer drugs across cell membranes against concentration gradient with the consumption of ATP (28), such as mitoxantrone, methotrexate, topotecan, SN38, and flavopiridol (29–31). ABCG2 expresses in normal tissues to protect the organism against toxic xenobiotics (32). Studies also showed overexpression of BCRP in certain drug resistant cell lines and tumors and associated with clinical drug resistance and lower survival rate (26, 33, 34). BCRP functions as ABC drug transporters to efflux a wide spectrum of chemotherapeutic agents, thereby conferring a multidrug-resistant phenotype (35–39). Increasing modulators of ABCG2 were developed, however, led to disappointing outcome in clinical trials (34, 40).

Previous studies have found that hormone nuclear receptors are important determinants of BCRP expression. It has been found that peroxisome proliferator-activated receptor γ (PPARγ) directly regulates the transcription of ABCG2 through binding to the PPAR response elements (PPARE) in the promoter (41). In BCa, BCRP expression was regulated by estrogen though directly binding of steroid receptors to ERE/PRE in the promoter region (22, 42–44). Our findings add significantly to the understanding of how BCRP upregulated during cancer development.

Previous studies have revealed the controversial role of TRPS1 in BCa progression. TRPS1 may cooperate with different co-transcriptional regulators to function as a transcriptional repressor or activator with regard to cell type, microenvironment, stages of development, or pathological conditions (16). TRPS1 behaved as a transcriptional repressor though recruitment of Ikaros family (45). Recent studies revealed epigenetic alteration at TRPS1 binding site, including H3K27ac and H3K27me3 (46, 47). TRPS1 is required to repress spurious binding of ER for removal of histone acetylation (48). The underlying mechanism of TRPS1 upregulation of BCRP remains unclear. Our results suggested that TRPS1 might regulate BCRP through transcriptional inhibition of miR-181a. In addition, TRPS1 affects sensitivity of drugs that are not typical substrate of BCRP. Besides BCRP, some other drug resistant mechanisms may also be involved in TRPS1 induced chemoresistance.

In conclusion, we have demonstrated that the expression of TRPS1 is correlated significantly with chemoresistance in BCa. Mechanically TRPS1 promoted BCRP gene expression and efflux transportation in BCa cell sublines. Our observations offer new perspectives for chemotherapy. Despite overexpression of TRPS1 usually predicts a better prognosis in BCa, chemoresistance may develop in patients with high TRPS1 expression. Further investigation involving multicenter clinical samples is warranted.

## Data Availability Statement

Publicly available datasets were analyzed in this study, these can be found in the NCBI Gene Expression Omnibus (GSE45255, GSE25055, GSE12791, and GSE27830).

## Ethics Statement

The studies involving human participants were reviewed and approved by Research Ethics Committee of Shandong University School of Medicine. The patients/participants provided their written informed consent to participate in this study.

## Author Contributions

PS and GZ contributed to the conception and design of the study. JH, HZ, and LL performed the experiments presented in the study. Data analysis was carried out by JH and BH. JH and PS were involved in writing the manuscript. All authors read and approved the submitted version.

## Conflict of Interest

The authors declare that the research was conducted in the absence of any commercial or financial relationships that could be construed as a potential conflict of interest.

## Supplementary Material

Figure S1 Validation of TRPS1 protein expression of indicated BCa cells by western blot analysis. **(A)** MDA-MB-231 **(B)** MCF-7.

Figure S2 TRPS1 correlated with drug sensitivity of BCa cells. **(A–C)** MDA-MB-231 cells were transfected with plasmids expressing TRPS1 (231-TRPS1) or empty plasmid (231-Vec). **(D–F)** MCF-7 cells were transfected with siRNA-targeting TRPS1 (MCF7-TS) or negative control (MCF7-nc). Cell viability and 50% inhibitory concentration (IC50) were assessed using the MTT assay after treatment of chemotherapeutic drugs. Cell viability was calculated relative to untreated controls. **(A,D)** Cisplatin; **(B,E)** 5-FU; **(C,F)** paclitaxel. Left panel: Cell viability. Right panel: 50% inhibitory concentration (IC50). Data represent averages from triplicates in a representative experiment, and their standard errors are depicted. ^*^*P* < 0.05, ^**^*P* < 0.01.
