# Supplementary figures and images for: TRPS1 Confers Multidrug Resistance of Breast Cancer Cells by Regulating BCRP Expression
Source: Front Oncol. 2020 Jun 30;10:934. doi: 10.3389/fonc.2020.00934 (PMC7338551; doi:10.3389/fonc.2020.00934)

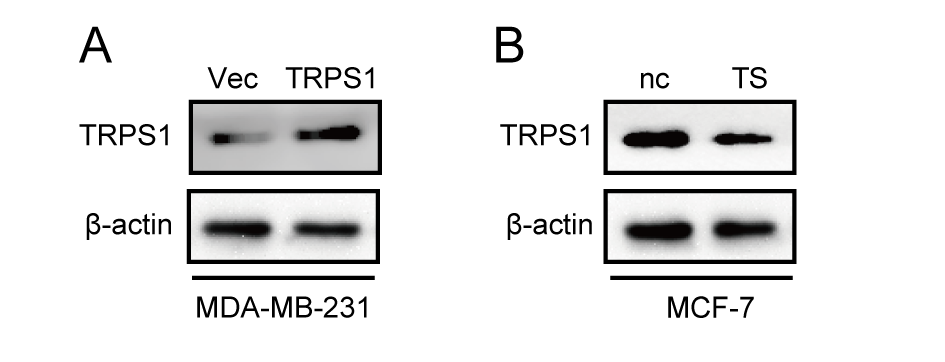

Supplement: Figure S1 — Validation of TRPS1 protein expression of indicated BCa cells by western blot analysis. (A) MDA-MB-231 (B) MCF-7. [file Image_1.tif]

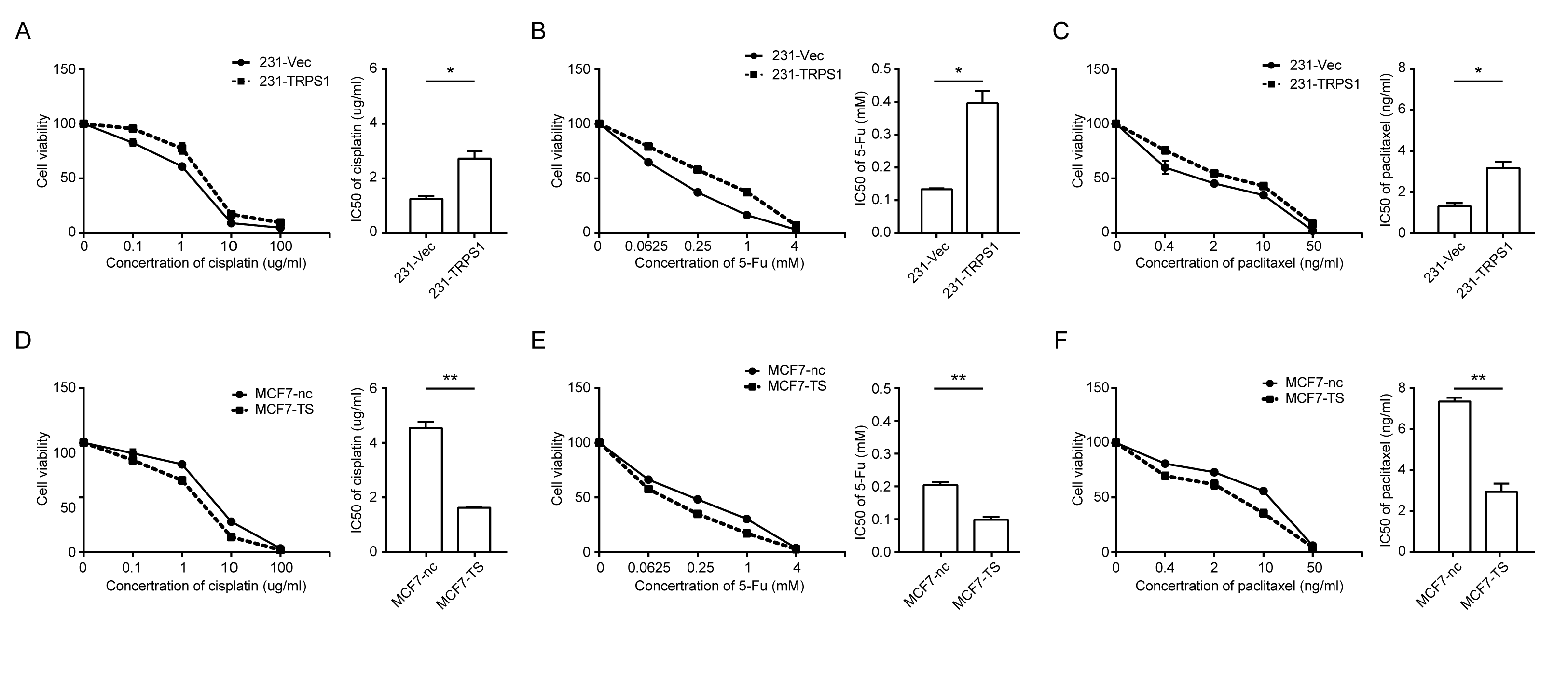

Supplement: Figure S2 — TRPS1 correlated with drug sensitivity of BCa cells. (A–C) MDA-MB-231 cells were transfected with plasmids expressing TRPS1 (231-TRPS1) or empty plasmid (231-Vec). (D–F) MCF-7 cells were transfected with siRNA-targeting TRPS1 (MCF7-TS) or negative control (MCF7-nc). Cell viability and 50% inhibitory concentration (IC50) were assessed using the MTT assay after treatment of chemotherapeutic drugs. Cell viability was calculated relative to untreated controls. (A,D) Cisplatin; (B,E) 5-FU; (C,F) paclitaxel. Left panel: Cell viability. Right panel: 50% inhibitory concentration (IC50). Data represent averages from triplicates in a representative experiment, and their standard errors are depicted. *P < 0.05, **P < 0.01. [file Image_2.tif]
